# An Online SBAS Service to Improve Drone Navigation Performance in High-Elevation Masked Areas

**DOI:** 10.3390/s20113047

**Published:** 2020-05-27

**Authors:** Hyojung Yoon, Hyojeong Seok, Cheolsoon Lim, Byungwoon Park

**Affiliations:** 1 School of Aerospace Engineering, Sejong University, Seoul 05006, Korea; hyovov@sju.ac.kr (H.Y.); csleem@sju.ac.kr (C.L.); 2 Security/ANS Certification Department, Aviation Certification Division, Korea Institute of Aviation Safety Technology, Incheon 22581, Korea; 3 Unmanned Vehicle Advanced Research Center, Korea Aerospace Research Institute, Daejeon 34133, Korea; hjseok317@kari.re.kr

**Keywords:** GNSS, SBAS, drone, DGPS, accuracy, integrity, protection level, RTCM

## Abstract

Owing to the high demand for drone operation in high-elevation masked areas, it is necessary to develop a more effective method of transmitting and applying Satellite-Based Augmentation System (SBAS) messages for drones. This study proposes an onboard module including correction conversion, integrity information calculation, and fast initialization requests, which can enable the application of an online SBAS to drone operation. The proposed system not only improves the position accuracy with timely and proper protection levels in an open sky, but also reduces the initialization time from 70–100 s to 1 s, enabling a drone of short endurance to perform its mission successfully. In SBAS signal-denied cases, the position accuracy was improved by 40% and the uncorrected 13.4 m vertical error was reduced to 5.6 m by applying an SBAS message delivered online. The protection levels calculated with the accurate position regardless of the current location could denote the thrust level and availability of the navigation solution. The proposed system can practically solve the drawbacks of the current SBAS, considering the characteristics of the low-cost receivers on the market. Our proposed system is expected to be a useful and practical solution to integrate drones into the airspace in the near future.

## 1. Introduction

Unmanned aircraft system (UAS) operations are rapidly increasing in number, technical complexity, and sophistication [1,2]. The growth in popularity and technical maturity of UASs (the so-called drones) are creating great expectations with respect to the benefits of replacing human labor and risks by the use of drones. The vision for fully integrating drones into the National Airspace System (NAS) implies that drones will operate harmoniously, side by side, with manned aircraft, occupying the same airspace and using many of the same air traffic management (ATM) systems and procedures [3]. Drone-specific standards and systems that supplement the already existing ones will ultimately be developed. However, as drones are also aircraft, the existing rules and infrastructure need to be applied to drones to a large extent [4].

Although there still remain many technical issues to be solved [5], one of the primary challenges facing the safe integration of drones into the civilian airspace is the availability and reliability of robust navigation that is sufficient to guarantee the sense-and-avoid capability. Most drone navigation systems on the civilian market rely primarily on the global navigation satellite system (GNSS) as the sole source of absolute position [6]. They should also include the ability to apply performance-based navigation (PBN) to operate in a similar manner to that of aircraft that rely on satellite positioning [7].

Considering the various advantages of the satellite-based augmentation system (SBAS) in terms of PBN support, this system could play a significant role in any future unmanned traffic management (UTM) framework in terms of providing the vital integrity and accuracy for drones [8]. Considering the philosophy of integrating drones harmonically into the NAS, that is to say, maintaining the infrastructure and standards that have been applied to the existing aircraft as much as possible, it is highly likely that drones could use the SBAS without major changes to the system construction or message structure. However, there is a high demand for drone operation in areas where civil aviation aircraft have not been used, such as urban canyons and mountainous areas. Thus, we need to review whether the SBAS can be used for drones without any structure change and, if necessary, provide solutions. In this paper, we consider that operation in high-elevation masked areas implies a remarkable difference that distinguishes drones from civilian aircraft in terms of GNSS positioning, and we discuss the application of the SBAS in such environments. However, another active research topic, as to whether the parameters applied to civil aircraft can also be applied to drones [9,10], is not included in the scope of this paper.

The remainder of this paper is structured as follows. In Section 2, the drawbacks of the SBAS for drone operation and the methodology to solve them are introduced. Section 3 describes the strategy to enable the proposed concept and verifies its feasibility for drone operation based on static test results. The effectiveness of the proposed system during the real operation of a drone in terms of improving accuracy, providing integrity information, and reducing the initialization time, is analyzed in Section 4. The discussions and conclusions are presented in Section 5.

## 2. Online SBAS Service Concept for Safe Integration of Drones in Urban Airspace

### 2.1. SBAS for PBN Operation

#### 2.1.1. SBAS Architecture and Segments

**Figure 1:**
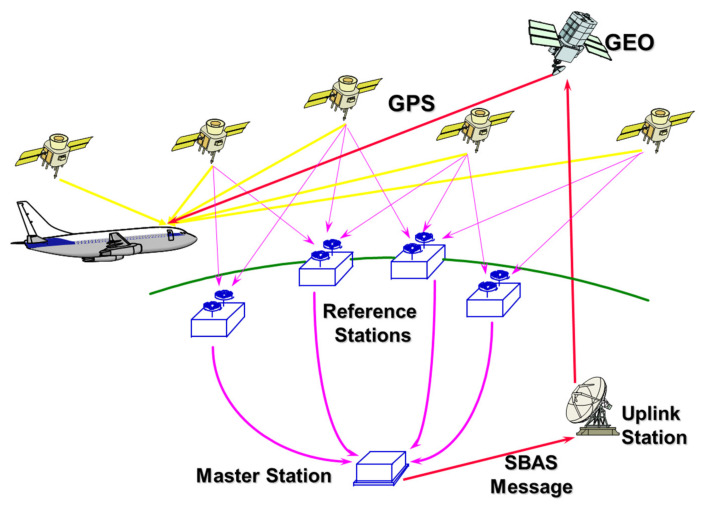
Satellite-Based Augmentation System (SBAS) architecture [14].

An SBAS is a civil safety critical system that supports wide-area augmentation using satellites that broadcast the augmentation information of integrity and correction. The SBAS architecture generally consists of a ground segment, i.e., a network of reference stations, master stations, and a geostationary (GEO) satellite control center, as well as a space segment [11,12] and a user segment, as illustrated in Figure 1. As all implemented SBASs, including the Wide Area Augmentation System (WAAS) in the United States, the European Geostationary Navigation Overlay Service (EGNOS) in the European Union, the Multifunctional Satellite Augmentation System (MSAS) in Japan, the GPS-Aided Geo-Augmented Navigation (GAGAN) system in India, and the future Korea Augmentation Satellite System (KASS) in Korea are operated by national programs [13], circular GEO satellites orbiting at the longitudinal orbital slots near the operating countries are used as satellite segments so that their service areas can cover the flight information regions (FIRs) of these countries.

The signal from the GEO satellite has a structure very similar to that of the GPS L1 C/A signal, with the same frequency, which enables GNSS avionics to receive the augmentation information without any additional communication channels. All the operating SBASs above comply with a common global standard, the Radio Technical Commission for Aeronautics (RTCA) DO-229 [15], which makes the systems compatible and interoperable so that SBAS-capable receivers can improve their navigation performance regardless of the coverage they are operating within. Owing to these advantages of the SBAS technology, not only the coverage and performance of the legacy ground-based navigation aids but also their capability, flexibility, and even cost-effectivity have been substantially increased, and PBN has finally become possible in most airspace areas.

#### 2.1.2. SBAS Message Format

**Figure 2:**
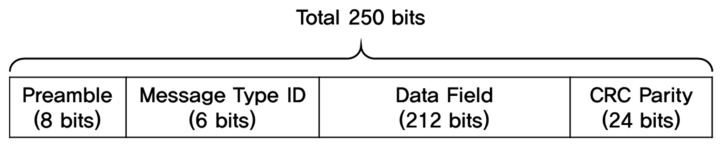
SBAS message structure [15].

**Figure 3:**
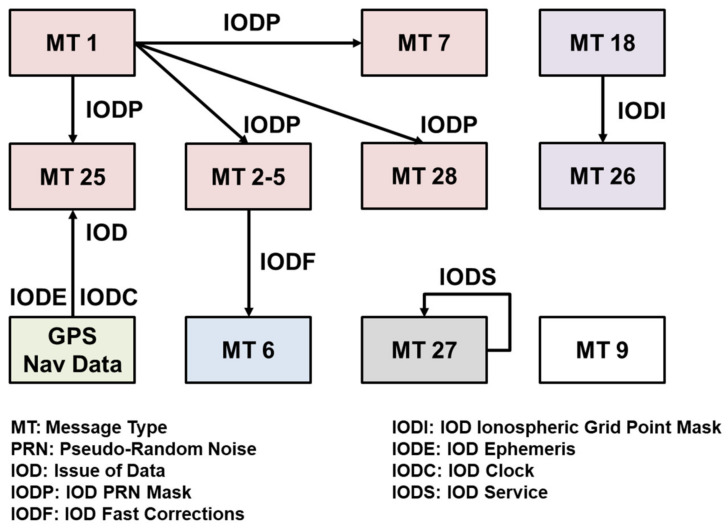
Interrelationship of the SBAS messages.

While each GPS satellite transmits navigation message at a speed of 50 bps, SBAS GEO satellites broadcast the signal at a transmission rate of 250 bps. The SBAS message structure consists of an 8-bit preamble, a 6-bit message type ID, a 212-bit data field, and 24-bit cyclic redundancy check (CRC) parity, as shown in Figure 2, and all the parameters for correction and integrity information should be included in the 212-bit data field [15] each second. To provide correction and integrity information for a wide area with a limited data bandwidth of 212 bits, the GEO satellite vectorizes and splits the messages into 64 message types (MTs) when transmitting them to the user segments, and the user receivers recombine the split messages to form correction and integrity information based on the interrelationship between the MTs, as illustrated in Figure 3.

Each message is broadcast at a dynamic rate based on its own update interval, as presented in Table 1, to utilize the bandwidth effectively. The WAAS messaging system specifies an update rate of 120 s and 300 s for ephemeris ionosphere corrections, respectively, while the fast corrections should be transmitted as often as every 6 s to meet the time-to-alert (TTA) requirement of the precision approach operation.

#### 2.1.3. SBAS Benefits in Drone Operations

Although the SBAS was originally designed and developed for civil aviation, it is easy to utilize the SBAS in non-aviation applications such as maritime, railroad, car navigation, and smartphones, owing to its simplicity, capability, and cost-effectivity. For example, the SBAS-augmented GNSS is replacing the maritime differential GNSS (DGNSS), which is expected to lead to the discontinuation of the differential GPS (DGPS) sites of the United States Coast Guard (USCG) by the end of 2020 [17]. Currently, around 80% of the GNSS receivers in use and available on the market are SBAS-enabled [18], which means the SBAS-capable function is inherent in most receivers, even those of low cost.

An SBAS message transmitted via the GPS-like L1 frequency signal is also beneficial to save the battery energy of a drone and extend its flight time as it enables the improvement of its navigation performance without additional communication channels and receipt of correction messages from a ground control system. It also enhances the simplicity of the system configuration.

The integrity information of an SBAS is a basis of separation from other aircraft, and will also be valid for drones when they become integrated into the civil aviation airspace in the near future. The protection level (PL) and assured position are expected to enable drones to be flown beyond the visual line of sight (BVLOS) so that they can sense and avoid other aircraft in a timely and proper manner. Integrity information is expected to play a key role in achieving the ultimate goal of drones entering the airspace, and the SBAS is definitely the best, easiest, and most realistic way to attain this.

To take advantage of these SBAS advantages, many drones are equipped with SBAS-capable GNSS modules [19,20] within SBAS-available regions. However, the SBAS functions of most GNSS modules in drones are for providing sub-meter accuracy, not for providing protection levels, since the module has been developed for general purposes, not for aviation.

#### 2.1.4. SBAS Drawbacks in Drone Operations

Despite the various benefits described above, there are several drawbacks when applying the SBAS to the drone navigation that might threaten its operation. The first critical drawback of the SBAS is its inability to service when the GEO is blocked by any obstacle. A drone should fly within the GEO-visible area to receive SBAS messages continuously.

The elevation angle (*E*) of the GEO at a point of latitude of *φ* and longitude of *λ* is obtained by Equation (1) [21]

(1) $$E=cos^{-1}\left\{\left(R+h\right)sin\beta /d\right\}$$

where cosβ=cosφcosλ, $d=\sqrt{R^{2}+{\left(R+h\right)}^{2}-2R\left(R+h\right)cos\beta }$, and *R* and *h* are the radius of the earth and altimeter of the GEO, respectively.

**Figure 4:**
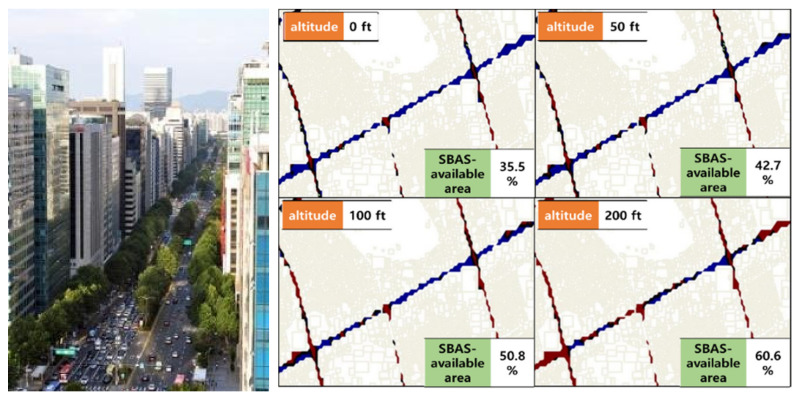
Urban canyon of Teheran-ro (**left**) and SBAS availability in Gangnam-gu, Seoul (**right**).

Assuming that the KASS GEO satellite will be located at the longitude of 143.5°, its elevation angle within the Korean flight information region (FIR) is only 42° to 45°, which implies that the SBAS can improve the drone’s navigation performance only when there are no obstacles blocking the GEO direction with an elevation of 45° or more. Simulation results based on three-dimensional building information from our previous work [22] demonstrated that drones flying at an altitude of 15 m or lower obtained benefits from the SBAS at 43% or less of the area of Gangnam-gu, Seoul, South Korea due to the high-rise buildings (Figure 4). Even if drones climb to an altitude of 60 m or more, it is not possible for them to obtain an SBAS-assured position (red dot in Figure 4, right) when they are flying in 40% of the urban area. The higher the latitude the drones are operating in, the less visible the SBAS GEO satellite is. At a latitude of 65°, a GEO satellite has an elevation angle of only around 17° at most, and at 75°, it is approximately 6° or less. Therefore, the condition of the line of sight direction to GEO would shrink the drone’s operating area.

The SBAS messaging system with various MTs and their own update intervals enables the transmission of wide area correction and integrity information at a low-rate datalink of 250 bps. However, it forces drones to wait up to 300 s of the ionospheric update interval to fully apply SBAS augmentation information. Considering that the endurance time of close-range unmanned aerial vehicles (UAVs) is 20 to 45 min [23], it is clear that an initialization time of 5 min would significantly hamper the entire mission. Moreover, there should be significant restrictions on mission execution or maneuvering during the initialization time, as drones need to receive the SBAS message for 300 s carefully and continuously.

Lastly, a large share of the GNSS receivers in the market apply the SBAS correction information but not integrity information, and this is one of the reasons why the integration of drones into the manned airspace is still a distant possibility.

### 2.2. Necessity of Online SBAS to Integrate Drones into Airspace

Whereas the full dependence on GEO improves the simplicity of the SBAS navigation equipment, there is a trade-off as it also limits the available area of drone operation. To enable the drone to perform its mission even in the GEO-invisible area, it is necessary to provide the SBAS message using a communication channel. As most drones performing missions already have a communication channel for control and data transmission from/to the ground control station (GCS), the complexity of the system does not increase due to the transmission of online SBAS messages. In addition, the SBAS data size of 250 bit per second is not large enough to affect the existing communication traffic.

An online SBAS service for seamless navigation in the GEO-invisible area can also contribute to shortening the SBAS initialization time. It is a similar way to how A-GPS shortens the time-to-first-fix time of smartphones by providing ephemeris information online [24]. The GCS can stack the latest information of each MT of the SBAS in the storage, and it can provide a group of the stacked messages of all the MTs to a drone if it requests a set of messages for fast initialization. Once the drone has received a full set of SBAS messages, it can obtain accurate positions continuously with their PLs, with only 250 bits of data for every second from the GCS as normal.

Even though GNSS receivers with multi-frequencies have recently been implemented in some deployed drones [25], only L1 GPS measurements are considered for this study because the current SBAS provides augmentation information for L1-only navigation. Code and carrier observables of the SBAS can increase the availability of navigation signals in urban areas; however they might also decrease the accuracy and integrity due to their high noise and frequent loss of lock [26]. Therefore, we utilized the online SBAS service in this study as a sub-meter accuracy augmentation system for GPS L1 code measurements, not for a ranging source.

## 3. Strategy to Enable Online SBAS Service for Drone Operation

### 3.1. Converting the SBAS for Radio Technical Commission for Maritime Services (RTCM) Correction

To describe the strategy to improve the accuracy of drones by the online SBAS service, this study considers the most widely deployed DGPS receiver, the U-blox 6 series module. This module supports both DGPS and SBAS, and we can confirm that their correction has been applied to the receiver through a flag of the position output, such as the National Marine Electronics Association (NMEA) data [27]. SBAS augmentation is possible only by receiving the SBAS L1 frequency signal transmitted from the GEO, whereas feeding the SBAS message to the receiver through its input port is not a possibility. The only way to enable DGPS through the input port is feeding Radio Technical Commission for Maritime Services (RTCM) correction. Thus, it is impossible to apply the online SBAS presented above as it is.

**Figure 5:**
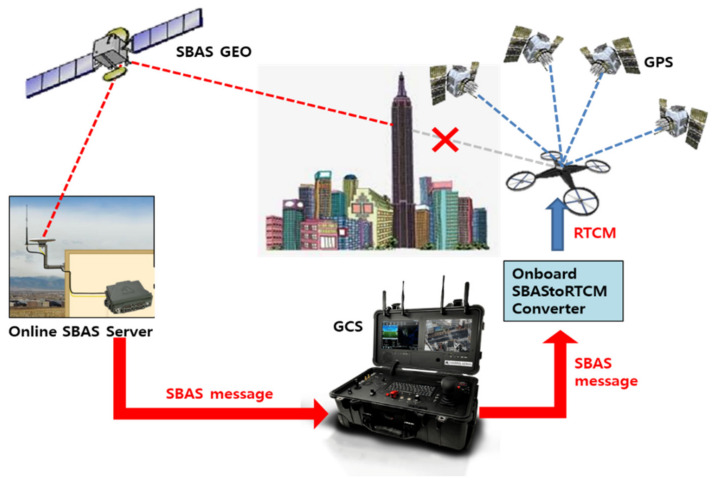
Online SBAS service concept for drone operation.

It is not appropriate for the GCS to provide the RTCM message instead of the SBAS because it is necessary to install a physical reference station connected to the GCS due to the DGPS coverage of 150 km. Meanwhile, if an online SBAS server is installed in an open sky region where GEO is visible, SBAS messages can be successfully delivered to the drones from the GCSs without any physical reference station, as shown in Figure 5, regardless of the locations of the GCSs and number of GCSs connected to the server. Given that most GNSS receivers of drones cannot decode and interpret the transmitted SBAS message through the input port, it should be converted into an RTCM message by the onboard module.

We performed an experiment to verify if the performance improvement from the DGPS converted from the SBAS message was equivalent to that of the SBAS by comparing the pseudo range correction (PRC) of the RTCM message with the range correction (RC) of the SBAS. We obtained SBAS messages for MSAS (PRN 129) on 21 May 2017 from the Server to Retrieve and Export Navigation Data (SERENAD) FTP server and generated the fast correction (FC), long-term correction (LC), and ionospheric correction (IC). Assuming that a drone was near YOND station (latitude of 35° and longitude of 129°), the RC for the *i*-th satellite was calculated from Equation (2) at the onboard module in the drone.

(2) $$RC^{i}=FC^{i}+LC^{i}+IC^{i}+TC^{i}$$

where TC represents the tropospheric delay.

The PRC was obtained by decoding the RTCM version 2.3 MT 1 from the YOND national DGPS (NDGPS) reference station (RS). The PRC was generated by subtracting the pseudo-range (*ρ*) from the estimated distance ($\hat{d}$), and the clock bias of a satellite ($\hat{b}$) and receiver ($\hat{B}$) as described in Equation (3).

(3) $$PRC^{i}=\left({\hat{d}}^{i}-\rho^{i}-{\hat{b}}^{i}+\hat{B}\right)=-\left(\delta d^{i}+\delta b^{i}+I^{i}+T^{i}\right)$$

where δd, δb, *I* and *T* are the GNSS errors of ephemeris, satellite clock, ionosphere, and troposphere, respectively.

**Figure 6:**
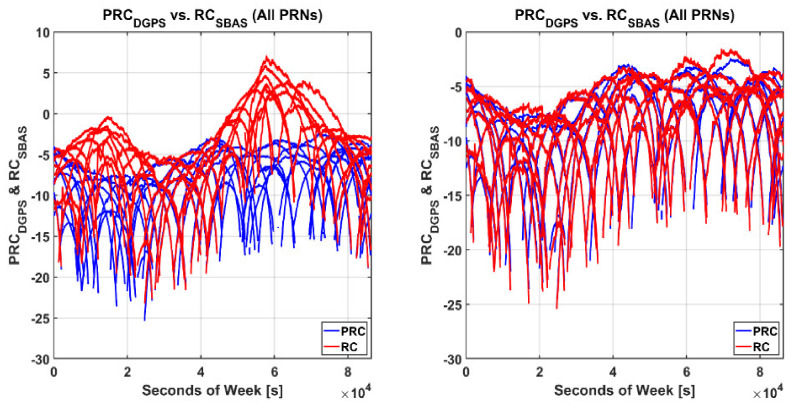
Comparison between pseudo range correction (PRC) and range correction (RC) before (**left**) and after removing bias (**right**).

Although Equations (2) and (3) are theoretically equivalent, the left plot of Figure 6 shows that there is a big difference between PRC and RC. This difference was due to the offset between the clock bias estimated from the SBAS reference station network and that of the NDGPS RS. After removing this bias between the two systems using Equation (4), the sets of corrections became identical, as represented in the right plot of Figure 6. The root mean square (RMS) of errors between the two sets of corrections was less than 0.5 m.

(4) $${\tilde{RC}}^{i}\left(t\right)=RC^{i}\left(t\right)-bias\left(t\right)$$

where $bias\left(t\right)=mean\left(\vec{RC}\left(t\right)-\vec{PRC}\left(t\right)\right)$ for each epoch t. $\vec{RC}$ and $\vec{PRC}$ are the sets of RC and PRC for all the visible satellites, respectively.

Using the relationship between the range domain and position domain correction based on DGPS-CP (correction projection) [28], we can compute the position corrections ($\delta \vec{x}$) by DGPS and SBAS using Equation (5).

(5) $$\left.\begin{array}{c} \delta {\vec{x}}_{SBAS}={\left(H^{T}\cdot H\right)}^{-1}\cdot H^{T}\cdot \vec{RC}\\ \delta {\vec{x}}_{DGPS}={\left(H^{T}\cdot H\right)}^{-1}\cdot H^{T}\cdot \vec{PRC} \end{array}\right\}$$

where H is an observation matrix in a local east-north-up (ENU) frame.

**Figure 7:**
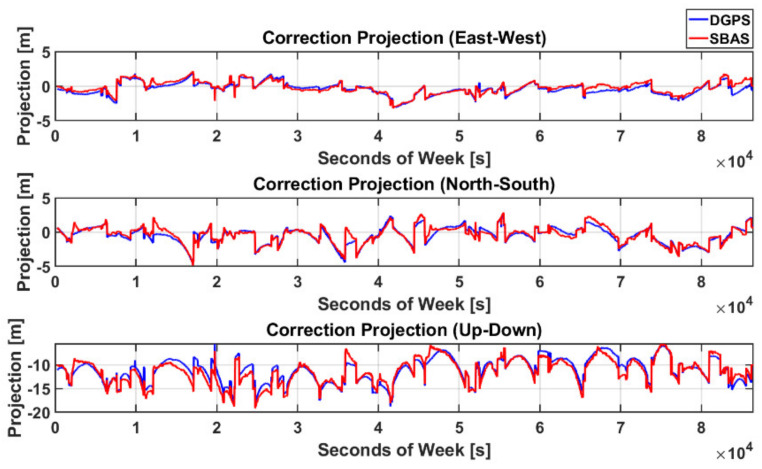
Projection of PRC and RC to the position domain.

As the above RC and PRC have almost the same value, it is obvious from Figure 7 that the position correction vectors of DGPS and SBAS that moved the stand-alone GNSS position closer to the true value were almost the same. The difference between $\delta {\vec{x}}_{SBAS}$ and $\delta {\vec{x}}_{DGPS}$ was so small that the horizontal and vertical RMS values were 0.65 and 1.0 m, respectively, representing a typical DGPS performance level. This result implies that the performance difference between the two techniques was negligible.

**Figure 8:**
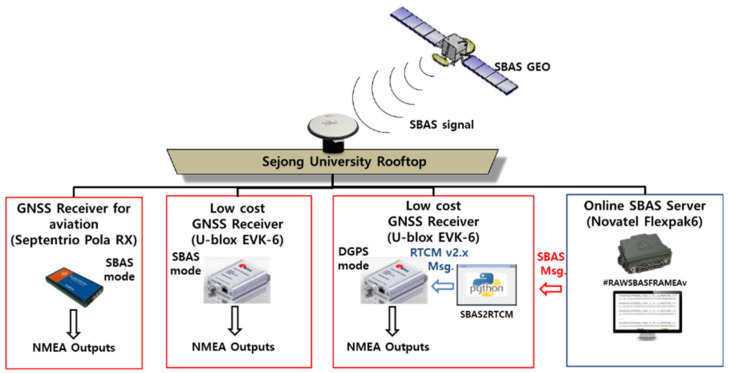
Static test system construction.

Based on the results obtained thus far, we conducted a preliminary feasibility test to apply the correction transmitted via internet from the online SBAS service to a low-cost receiver, U-blox EVK-6. To assess the accuracy performance of our suggested system, we compared the results with a target high-end SBAS-supporting receiver, Septentrio PolaRx3, which had been used for the EGNOS flight testing. We constructed an SBAS static test set, which consisted of two U-blox EVK-6s, one Pola RX, and one Novatel Flexpak6 for the online SBAS server operation in Figure 8. The Pola RX and one EVK-6 were set to the SBAS mode that corrected their positions using the L1 GEO signal and inherent SBAS function, and one Novatel Flexpak 6 and the other EVK-6 as our suggested system were also connected with them to the same antenna via a splitter. We developed an SBAStoRTCM module in Python that converted the SBAS message delivered in #RAWSBASFRAMEA format from the online SBAS server of Flexpak 6 to the RTCM format. The SBAStoRTCM module calculated RCs for the satellites in view from the NMEA GPGSV by using the rover’s approximate latitude, longitude, and height information of the NMEA GPGGA and transmitted SBAS binary message, and then generated RTCM corrections by encoding the RC to the PRC of MT 1. The EVK6 as the rover system should be set as the DGPS mode and should obtain the RTCM-converted message via its input port to differentiate its position errors. We could confirm the validity of our system by comparing the results with those of EVK-6 with the SBAS mode, and the feasibility of the online SBAS service in low-cost receivers was evaluated through a performance comparison with Pola RX. The static test was conducted from 09:34 to 10:34 (UTC) on 25 April 2018.

**Figure 9:**
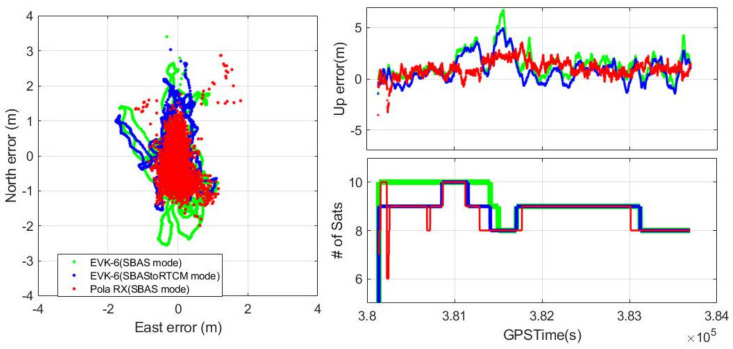
Static test positioning results.

Figure 9 shows the horizontal and vertical error variations of the three receivers and the number of satellites that were used for their position calculation. The number of active satellites of the two EVK-6s was the same from 309,053 to 381,112 s GPSTime and for the last 33 min of the test, and their difference for the rest of the time was just one. During the period when both receivers used the same satellite set, their position differences were small despite using different positioning modes, and the RMSs of the positioning difference between the two modes were 0.73 and 0.86 m horizontally and vertically, respectively.

The overall performance of the receivers is presented in Table 2. The three-dimensional error RMS values of the two EVK-6 receivers were 2.1 and 1.9 m, respectively, and the difference was less than 10% of the performance. Even though the RMS of EVK 6 with the SBAStoRTCM mode was slightly greater than that of Pola RX by 0.5 m, its maximum errors, 2.7 m (horizontal) and 4.9 m (vertical), were smaller than the 95% accuracy requirements of the SBAS Approach with Vertical guidance(APV)-II, which are 16 and 8 m, respectively. Therefore, it is reasonable to improve the accuracy performance of a low-cost receiver using online SBAS during drone operation in a high-elevation masked area.

### 3.2. Calculating and Providing Protection Levels of Drones

Most low-cost receivers apply SBAS augmentation messages to improve accuracy, but do not use integrity information to provide the PL or a timely and proper alert. The PL is essential for guaranteeing the separation distance when a number of aerial vehicles are in operation. Thus, receivers for drones must include the ability to compute the PL to be integrated into the airspace [29].

The SBAS protection level equations were based upon the observation that the error sources are approximately Gaussian and that an inflated Gaussian model can be used to conservatively describe the positioning errors [30,31]. The conservative variance of the individual pseudorange residual (σ) was formed by the sum of the variances for the fast long-term satellite correction ($\sigma_{flt}$), the interpolated grid ionospheric vertical error (GIVE) values converted to slant ($\sigma_{UIRE}$), the slant tropospheric error ($\sigma_{trop}$), and a fixed function of elevation for airborne noise and multipath ($\sigma_{air}$), as shown in Equation (6). The last term, $\sigma_{air}$, for different performance levels of GPS receiver technologies [32,33] is described in Appendix J [15]. We used the Ground Accuracy Designators (GAD)-A level model because the model for commercial low-cost receivers has not been determined yet.

(6) $$\sigma^{2}={\sigma_{flt}}^{2}+{\sigma_{UIRE}}^{2}+{\sigma_{trop}}^{2}+{\sigma_{air}}^{2}$$

The pseudorange variance of Equation (6) was inverted to be placed on the diagonal elements of the weighting matrix, W, and was combined with the geometry matrix in the local ENU frame, H of Equation (5), to form the covariance of the position estimate (P).

(7) $$P={\left(H^{T}WH\right)}^{-1}$$

When naming each component of P as Equation (8)

(8) $$\left[\begin{array}{cccc} {d_{east}}^{2} & d_{EN} & d_{EU} & d_{ET}\\ d_{EN} & {d_{north}}^{2} & d_{NU} & d_{NT}\\ d_{EU} & d_{NU} & {d_{up}}^{2} & d_{UT}\\ d_{ET} & d_{NT} & d_{UT} & {d_{T}}^{2} \end{array}\right]=P$$

the horizontal (HPL) and vertical protection level (VPL) are given by

(9) $$\left.\begin{array}{c} HPL_{SBAS}=K_{H.PA}\cdot d_{major}\\ VPL_{SBAS}=K_{V}\cdot d_{up} \end{array}\right\}$$

where $d_{major}$ is $\sqrt{\frac{{d_{east}}^{2}+{d_{north}}^{2}}{2}+\sqrt{{\left(\frac{{d_{east}}^{2}-{d_{north}}^{2}}{2}\right)}^{2}+{d_{EN}}^{2}}}$ and $K_{H.PA}$, $K_{V}$ are 6.0 and 5.33.

**Figure 10:**
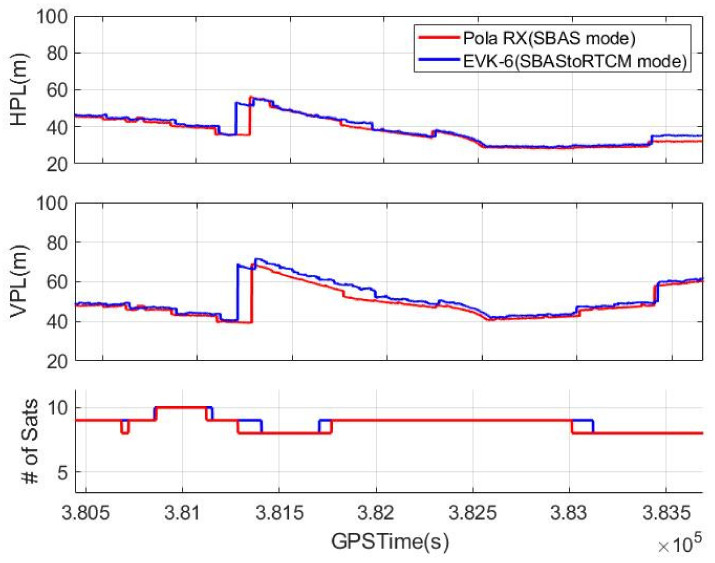
Protection level comparison (**top**: horizontal protection level (HPL), **middle**: vertical protection level (VPL), **bottom**: number of active satellites).

The HPL and VPL calculation modules for EVK-6 were added to the experimental configuration in Figure 8, and our calculated PLs were compared with the values provided by the Pola RX receiver. Figure 10 shows the result of this comparison, and the difference between the PLs from the two systems was so small that their RMSs were only 1.02 m horizontally and 2.16 m vertically. As is well known, EVK-6 itself does not have any function to calculate the PL, and thus it is not shown in Figure 10. 

### 3.3. Modifications of the Ground Facility to Shorten SBAS Initialization Time

A long initialization time of up to 300 s is another critical drawback to be solved by the suggested online SBAS service. When the initialization process is required for a drone, our onboard module requests a quick initialization mode to the online SBAS server, and the server provides the drone with the stacked up to date SBAS messages at once. The onboard module converts SBAS corrections to the RTCM message and computes PLs as soon as it receives a set of the latest SBAS messages, and then the drone can compute its accurate position assured by the computed PLs just 1 s after sending the quick initialization mode request. Once the drone obtains an accurate and guaranteed position, its mode changes to a normal operating mode and it can acquire continuous positions with PLs, with only 250 bits of data for every second from the GCS.

**Figure 11:**
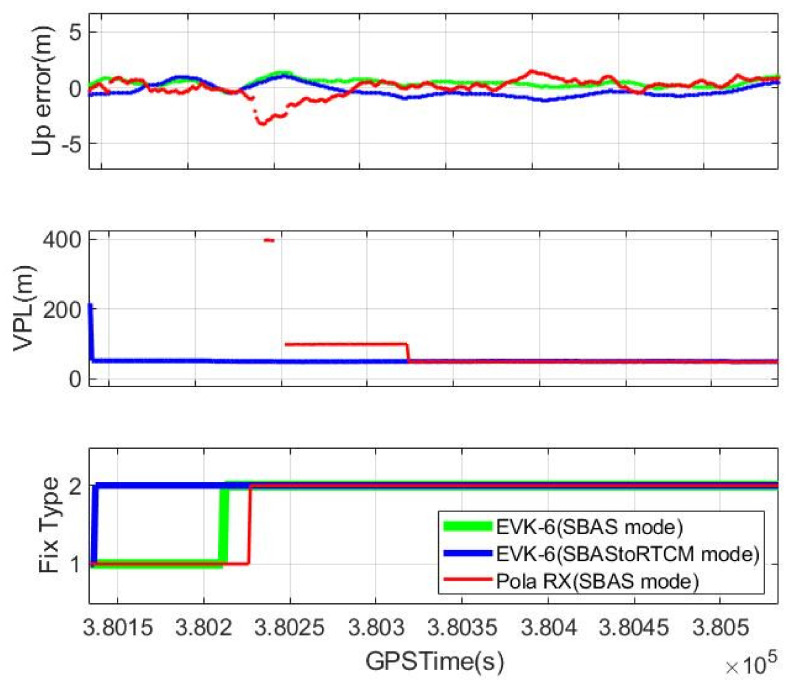
Initialization time comparison (**top**: vertical error, **middle**: VPL, **bottom**: fix type).

Figure 11 shows the effect of the quick initialization mode. It represents the vertical errors, their VPLs, and the fix types from the NMEA outputs of the three receivers during the initial 400 s. The fix type, which indicates stand-alone for 1 and DGPS for 2, denotes when the SBAS reception was completed. It took 75 and 90 s for the EVK-6 and Pola RX to switch to the DGPS mode by receiving the L1 SBAS signals from GEO, whereas the EVK-6 with the onboard module acquired its DGPS solution just 1 s after requesting the quick initialization mode. The vertical errors of the other two receivers were not large despite of the stand-alone mode, and the presumed reason is that much of their error was mitigated by error models such as the Klobuchar and tropospheric models. This assumption is reasonable because the position errors of EVK-6 in the SBAS mode became the same as those of our system immediately after switching to the DGPS mode. In the case of Pola RX, the errors increased immediately after its mode changed to DGPS, because Pola RX did not obtain SBAS messages for all the visible satellites at the time, and only six satellites among them were used for the DGPS positioning, as shown in Figure 9.

The PL calculation of Pola RX took longer than solving the DGPS position. Our system calculated a VPL of 50 m just after receiving the quick initialization message from the server, but Pola RX did not provide any VPL for precision approach for the initial 150 s. The initially calculated PL value was even much larger than ours, and it took 232 s to calculate a proper PL.

## 4. Field Test Results

### 4.1. System Configuration

**Figure 12:**
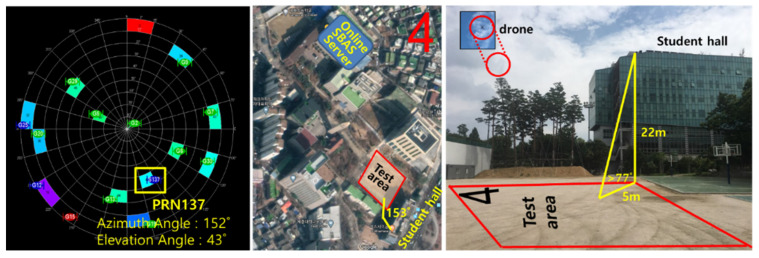
Experimental location and environment (**left side**: direction of PRN 137, **middle**: map of test area, **right side**: view of test area).

We constructed a real dynamic configuration in which a drone could not receive the SBAS signal from the GEO due to obstacles such as nearby buildings. The PRN 137 GEO, for which Japan’s MSAS signal is receivable in the Korean region, was visible from the azimuth of 152° and elevation of 43° in Seoul. A student hall in a six-story building was located in the southwest of the sports ground of Sejong University, where a drone was flying for the dynamic test. As the building’s height was 22 m, the drone placed on the ground horizontally 5 m apart from the building could not see the PRN 137 GEO, as shown in Figure 12. A GNSS antenna for the online SBAS server, constructed on the rooftop of the Chungmu-gwan building, was located 320 m away from the drone test area.

**Figure 13:**
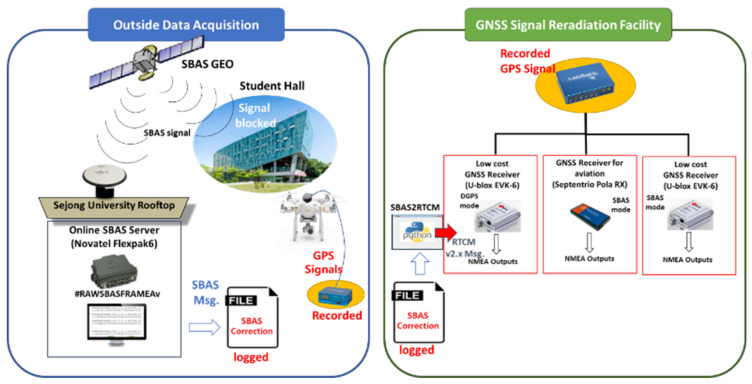
Field test replay set configuration.

A DJI Phantom 3 was used for the dynamic field test from 08:01:09 to 08:12:19 UTC on 18 October 2018. As it allows little weight and space for a payload, it was not possible to mount all the test sets described in Figure 8. Instead, we acquired all GNSS signals including GPS and SBAS during the drone’s flight using a Labsat 3 device that could record and reradiate the RF signal. Labsat 3 captured GPS signal during the drone’s flight and transmitted the signal to receivers in the office. During the signal collecting test, we also logged the #RAWSBASFRAMEA SBAS correction transmitted from the online SBAS server into a file. After acquiring all the GNSS signals and SBAS correction, we constructed a GNSS signal reradiation facility, as shown in Figure 13. We were able to replay the real time process of the online SBAS and SBAStoRTCM module by feeding a timely correction message to the system using the method presented in our previous study [34]. As shown in Figure 8, Pola RX and EVK-6 were set to the SBAS mode and received the signals reradiated from Labsat, and the other EVK-6 was connected to the SBAStoRTCM module to operate in the DGPS mode. This test configuration enabled us to evaluate the performance of three different systems with a single drone flight.

### 4.2. Dynamic Test Scenario

**Figure 14:**
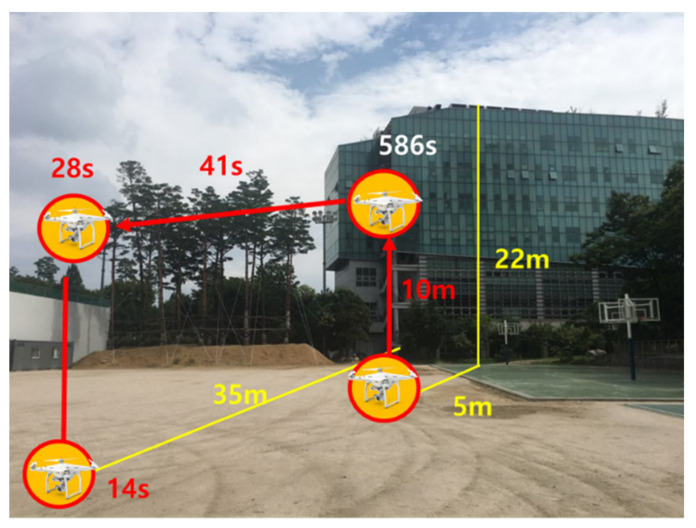
Dynamic test scenario.

To conduct a test on the situation where the SBAS signal was denied due to a nearby building, we set a scenario in which a drone took off from the ground near the student hall building, ascended to an altitude of approximately 10 m, moved to the SBAS-visible area, and then landed on the ground as represented in Figure 14.

The initial position of the drone was 5 m northwest of the building, and the height of the building was 22 m. No signal was transmitted with an elevation angle lower than 77° in the direction with an azimuth of 152°. Although it was hovering at the altitude of 10 m for 586 s after taking off, the signals at an elevation angle of 67° or less were still blocked. The PRN 137 GEO was not visible until it moved 8 m horizontally from the hovering point. The Pola RX and EVK-6 in the SBAS mode could receive the MSAS SBAS signal only for the last 1 min of the test. The true trajectory was computed by a post-processing tool, the Trimble Business Center (TBC).

### 4.3. Dynamic Test Results

**Figure 15:**
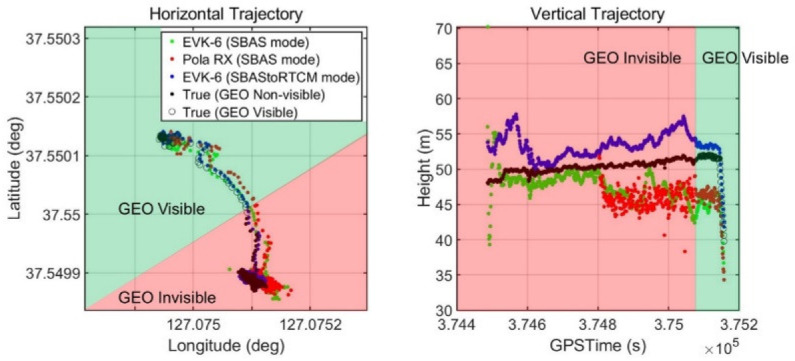
Drone flight trajectory and PRN #137 visibility.

According to the scenario described in Section 4.2, the horizontal and vertical true trajectories of the drones and the positioning results provided by the three devices in Figure 13 are shown in Figure 15. The points where the PRN 137 GEO was visible for the last 68 s are represented by bordered dots.

**Figure 16:**
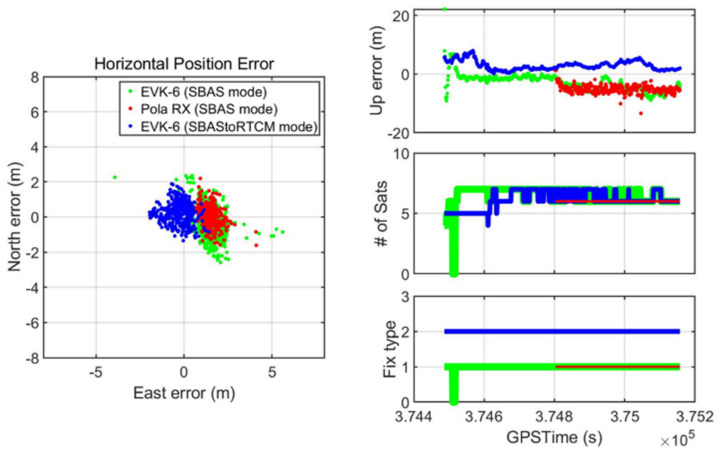
Dynamic test position accuracy (**left side**: horizontal error, **top right**: vertical error, **middle right**: number of active satellites, **bottom right**: position fix type).

The test results in Figure 16 show how our proposed system improved the drone’s navigation performance in terms of accuracy when the SBAS signal was denied by the actual building. Our developed system immediately changed its mode to DGPS and provided the error-mitigated positions, while the other EVK-6 in the SBAS mode eventually failed to provide DGPS position despite moving to the point where the GEO satellite was visible. For the receiver, 68 s was not enough time to accumulate the SBAS message transmitted at a low rate of 250 bps and generate a correction from them. The RX did not even provide a stand-alone position when it was near the building for the first 317 s.

The overall performance of the receivers for the dynamic test under the SBAS-denied area is presented in Table 3. The performance of the two receivers that could not properly apply the SBAS message was similar despite the differences between them. The average horizontal and vertical errors of the two devices were 1.5 to 1.6 m and −4.6 to −5.1 m, respectively, and their three-dimensional error RMS values were 5.3–5.6 m. Owing to the onboard module that applies the SBAS message transmitted from the online SBAS server, the navigation performance improved by more than 40%. Thus, the suggested system could achieve average horizontal and vertical errors of 0.5 m and 2.9 m, respectively, and its RMS was reduced to 3.2 m. In particular, the horizontal and vertical errors of 4.4 and 13.4 m, respectively, were reduced to 1.6–5.6 m by utilizing the transmitted SBAS message.

**Figure 17:**
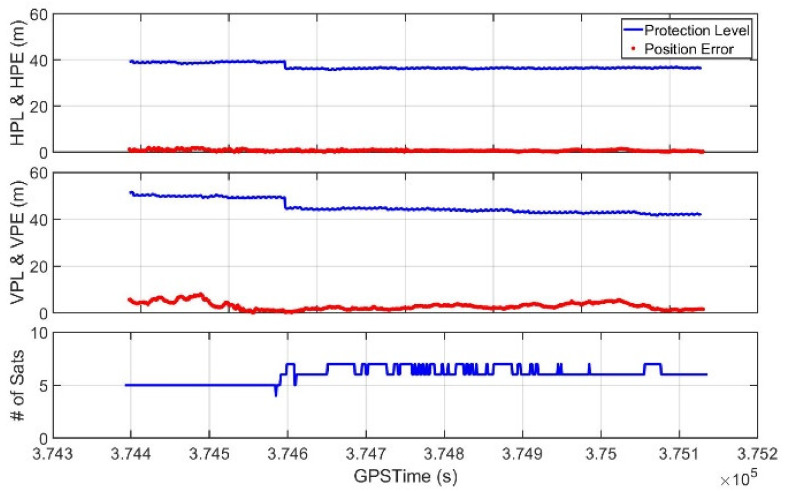
Position error and protection levels of the operating drone.

Our suggested system also provided proper PLs with the error-mitigated positions to ensure the level of trust to be placed on the solution. It could calculate the PLs as soon as the power of the receiver was turned on, which was confirmed from the static test result. Figure 17 shows that HPL and VPL were provided throughout the drone operation, and these values properly bounded the position errors.

**Figure 18:**
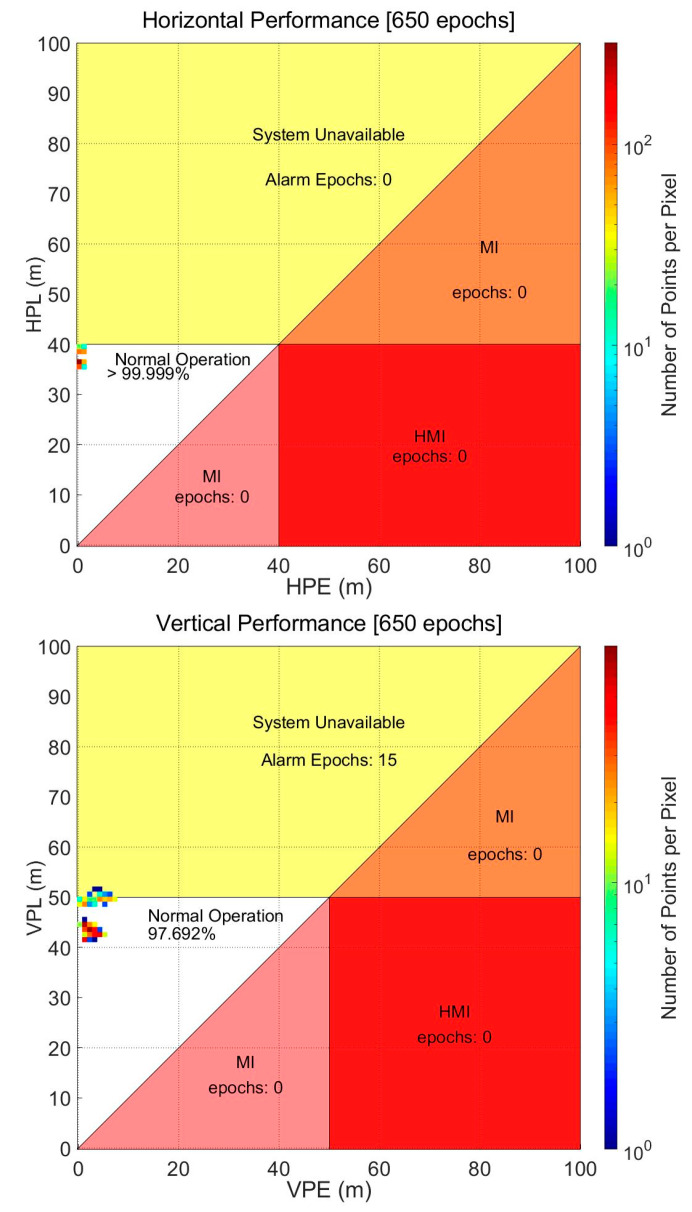
Horizontal (**left**) and vertical (**right**) Stanford plot for Multifunctional Satellite Augmentation System (MSAS) during drone operation (Approach with Vertical guidance, APV-I).

The VPLs at the beginning of the test, when the drone was flying near the student hall building, slightly exceeded 50 m and did not meet the APV-I requirement. However, after the initial 15 epochs, both HPLs and VPLs were smaller than the horizontal and vertical alert limits of 40 and 50 m, respectively, through the rest of the test. Therefore, the APV-I availability of this system was computed as 97.692% because the APV-I conditions were met for 654 epochs out of a total of 669 epochs, as shown in Figure 18. During the session, there were not found any misleading information (MI) hazardous misleading information (HMI) events.

## 5. Conclusions

It is vital to provide accurate positions with the timely and proper level of thrust for the drone’s operation BVLOS, which is essential to integrate drones into the civil airspace. As the SBAS can improve the accuracy and provide integrity information to aircraft regardless of an aircraft location, it is definitely a more suitable system for drones than any other GNSS augmentation system currently available in the aviation field. An aircraft can improve its navigation performance only after waiting for an initialization time of up to 300 s under the open sky. This condition is not a problem for conventional civil aircraft with large fuselage and long endurance times. However, it can be a serious limitation for drones. Given the high demand for drones in various areas and applications and their short operating time because of the limitations in battery technology, it is essential to calculate the accurate position and PL in a short time, even in high-elevation masked areas.

In this study, in order to solve these fatal drawbacks of the SBAS when applied to drones, we proposed an online SBAS service and an onboard algorithm for drones to apply the transmitted SBAS messages. Based on the theory that the RC of SBAS and the PRC of RTCM are equivalent, an SBAStoRTCM module was developed to improve the accuracy of the low-cost receiver, and the HPL and VPL were calculated and provided to the drone according to the RTCA standard procedures. Additionally, we added a fast initialization function by which the server could send the latest SBAS message stack at once when the drone requested to shorten the initialization time, enabling the drones to obtain the error-mitigated position and PLs in merely 1 s.

A static test using the algorithm and the implemented system demonstrated that even a low-cost U-blox EVK-6 receiver could improve its accuracy, making it comparable to the high-end Septentrio PolaRX, and verified that the difference in PLs calculated by the onboard module and Pola RX was only approximately 1 m. The fast initialization function shortened the waiting time from 230 s to 1 s to obtain sub-meter accuracy and proper PLs.

For a real-time test with the GEO signal blocked by a building, we conducted a dynamic test near a six-story building. The drone hovered near the building for 10 min and then moved to the GEO-visible area for the last 1 min. The period of 1 min was not sufficient for both the high-end receiver that supported civil aviation navigation and the low-end receiver to change their positioning mode from stand-alone to SBAS DGPS. However, our proposed system computed its DGPS position as soon as its power was turned on. The position accuracy was improved by 40% owing to the online SBAS system, and the uncorrected 13.4 m vertical error was reduced to 5.6 m. The PLs calculated with the accurate position indicated whether the drone’s current navigation solution was available in the APV-I category.

Therefore, the online SBAS server and the drone’s onboard module effectively improved the drone’s navigation performance, particularly in high-elevation angle masked regions such as mountains or urban canyons. The most important advantage of our system is that it is a practical way to solve the drawbacks of the current SBAS using off-the-shelf receivers on the market. We expect our proposed system to be a useful and practical solution for the integration of drones into the airspace in the near future.

## Figures and Tables

**Table 1:** Current Wide Area Augmentation System (WAAS) data update interval and usage [16].

Correction Type	Message Type (s)	Messages per Update Interval	Update Interval (sec)	Percent of Full Bandwidth
Satellite Mask	1	1	60	1.7%
Fast Corrections	2–4	2	6	33.3%
Fast Corrections (others)	5	0	60	0.0%
UDRE (User Differential Range Error) Update	6	0	6	0.0%
Fast Degradation	7	1	120	0.8%
Geo Navigation	9	1	120	0.8%
UDRE Degradation	10	1	120	0.8%
UTC/WAAS	12	1	300	0.3%
Geo Almanac	17	1	300	0.3%
Ionosphere Grid Mask	18	4	300	1.3%
Mixed Corrections	24	1	6	16.7%
Long-term Corrections	25	0	120	0.0%
Ionosphere Corrections	26	25	300	8.3%
WAAS Service	27	0	300	0.0%
UDRE modification	28	~10	120	8.3%
Total				71.2%

**Table 2:** Statistics of static test positioning results.

	Pola RX (SBAS)		EVK-6 (SBAS)		EVK-6 (SBAS to RTCM)	
	Horizontal	Vertical	Horizontal	Vertical	Horizontal	Vertical
RMS error	0.6808 m	1.2759 m	0.8494 m	1.9137 m	1.1072 m	1.5516 m
	1.4494 m		2.0906 m		1.9007 m	
Mean error	0.5934 m	1.0232 m	0.7043 m	1.4234 m	0.9599 m	0.8588 m
Maximum error	3.1261 m	3.5479 m	2.2237 m	6.7379 m	2.6533 m	4.9379 m

**Table 3:** Statistics of dynamic test positioning results.

	Pola RX (SBAS)		EVK-6 (SBAS)		EVK-6 (SBAStoRTCM)	
	Horizontal	Vertical	Horizontal	Vertical	Horizontal	Vertical
RMS error	1.7007 m	5.2982 m	1.8617 m	4.9505 m	0.6836 m	3.1570 m
	5.5645 m		5.2890 m		3.2302 m	
Mean error	1.5330 m	−5.1056 m	1.6567 m	−4.6357 m	0.5652 m	2.9423 m
Maximum error	4.4139 m	13.3900 m	3.4869 m	8.8930 m	1.5703 m	5.5960 m
Position available	Stand-alone: 52.7%		Stand-alone: 96.0%		Stand-alone: 100%	
	DGPS: 0%		DGPS: 0%		DGPS: 100%	
